# The use of polyurethane foam-sand mixtures in sandy embankment design- predicting seismic response using FEM, catastrophe theory, B-spline method, and artificial neural networks

**DOI:** 10.1016/j.heliyon.2024.e31719

**Published:** 2024-05-24

**Authors:** Omer Mughieda, Abdoullah Namdar, Wen Nie

**Affiliations:** a Department of Civil Engineering, Abu Dhabi University, Abu Dhabi, United Arab Emirates; b School of Civil Engineering, Iran University of Science and Technology (IUST), Narmak, Tehran, Iran; c State Key Laboratory of Safety and Health for Metal Mines, Maanshan, 243000, China

**Keywords:** Polyurethane foam, Sand, Embankment, Catastrophe theory, Seismic response, Failure mode

## Abstract

High-permeability sand cannot control the water that is stored behind an embankment. In addition, if clay cannot be provided within a reasonable distance of the embankment construction site, an alternative method must be found. The study proposes using a polyurethane foam-sand mixture to construct an impermeable embankment. The main purpose of the paper was to predict the seismic stability of the embankment. The nonlinear finite element models (FEMs) are applied along with artificial neural networks (ANNs), and this research method applied was performed to investigate the main objectives of the research. Catastrophe theory was used to predict the mechanism of differential displacement in the Y direction at selected points of the embankment model. For model smooth functions, the basis spline (B-spline) method was applied to simulate the catastrophe progression index value. Results revealed that the suitability of the polyurethane foam-sand mixture controls the acceleration, displacement, strain, and stress of the model at points selected in different parts of the embankment. Moreover, it was found that the deformation pattern of the model was related to the polyurethane foam-sand mixture ratios. Furthermore, the main contribution was that the seismic response of the embankment model could be improved with the right percentage of polyurethane foam added to the sand. Results were validated by referencing those available in the literature.

## 1 Introduction

Researchers mixed polyurethane foam with sand to improve permeability [1,2] and added it to municipal solid waste bottom ash to introduce a new material [3]. Also, the static and dynamic properties of the polyurethane foam-sand mixture were investigated [1,2]. The seismic response of the embankment was studied by considering the materials [4], geometry [5], and displacement of the model [6]. Despite this, a realistic seismic load needs to be applied to an impermeable embankment consisting of polyurethane foam-sand to investigate its seismic response. The novelty of this work is that the introduction of a polyurethane foam-sand mixture in the impermeable embankment design is a significant improvement that enhances its seismic resistance, making it more reliable and effective.

A geogrid system is proving effective in minimizing embankment displacement [6] as an example. Despite this, no consideration has been given to the embankment seismic designs, the permeability, or the availability of the materials.

Polyurethane foam was injected into the ground, and the silty soil permeability was reduced [7]. In addition, a polyurethane foam-water glass mixture was used to minimize subsoil water permeability [8]. By mixing polyurethane foam with soil, a composite material with new mechanical properties was created. This material has been used in highway pavement design and embankments. Therefore, embankments and highways constructed with these materials exhibit high strength, stiffness, and serviceability [9,10]. The polyurethane foam-sand mixture can produce an innovative, suitable construction material [1]. However, the low-level seismic stability of the embankment is a common reason for embankment failure [6,11,12]. Despite improvements in the mechanical properties of sandy soil due to the reinforcement with geofibers, high levels of permeability remain a problem [13,14]. In the strong earthquake [15,16], there was substantial damage and destruction to most of the embankment. As a result of cracks developing in the embankment [17], the subsoil of the embankment liquefied [18] and was deformed [19]; the fines contents of the soil caused the liquefaction in the Christchurch earthquake [20]; considering the liquefaction phenomenon validation was made using centrifuge test data [21]. In any case, the polyurethane foam-sand mixture did not crack due to changing groundwater levels and needs to be simulated using numerical simulation on a large scale in the simulated model.

In contrast to the tension cracks that may occur during construction or service in the core of the embankment [[22], [23], [24]], longitudinal cracks occur in its crest due to earthquakes [25,26]. An investigation of the cracking of the embankment clay cover was conducted using numerical simulation [4]. It was shown that a polyurethane foam-sand mixture minimized crack development in the embankment.

However, the impact of seismic acceleration on simulated embankments composed of polyurethane foam and sand has yet to be investigated. Additionally, using appropriate software to simulate the embankment subject to seismic loading is insufficient for engineering design. It is necessary to use suitable theoretical concepts and prediction methods to incorporate engineering decisions into embankment seismic design.

Many researchers have utilized ANNs to predict liquefaction [27], compaction properties of fine-grained soils [28], mixing soil [29,30], displacement at selected points of the clayey cover of the landfill model [4], soil thermal conductivity [31], and soil compaction [32]. In addition to geotechnical engineering, artificial neural networks have been applied in many fields of engineering [33], and to improve the accuracy of ANN predictions, genetic algorithms combined with ANNs [34] and deep learning combined with ANNs [35] have been applied. In this study, a catastrophe theory and a B-spline model were used to improve prediction quality.

Models based on catastrophe theory have been used to assess nuclear power plants [36], embankments seismically [37], and aluminum powder explosions [38]. The B-spline is a nonparametric density estimator for smoothing data sets and graphical representation [39], it was modified and was able to remove the oscillations [40,41]. In the region that lacks sufficient clay material for constructing the embankment, new construction materials are needed.

The main objectives and hypothesis of the present numerical simulation are as follows.

• Solving the problem of construction materials availability by using a polyurethane foam-sand mixture to construct impermeable embankments and subsoil.
• A combination of FEM, ANNs, catastrophe theory, and B-spline was applied to predict displacement, deformation, stress, and strain in the proposed model.
• Furthermore, the seismic stability of the model was examined using the differential displacement mechanism in the Y direction. This was compared to the multidirectional displacement mechanism.

### 1.1 Modeling

**Fig. 1:**
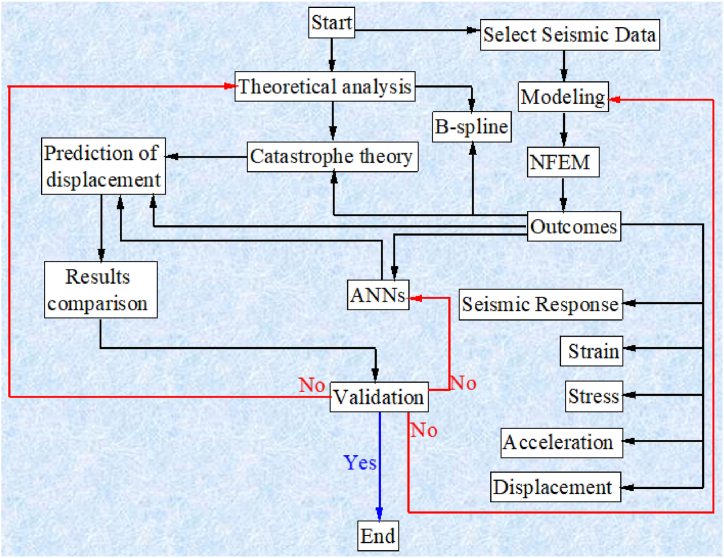
Study method.

**Fig. 2:**
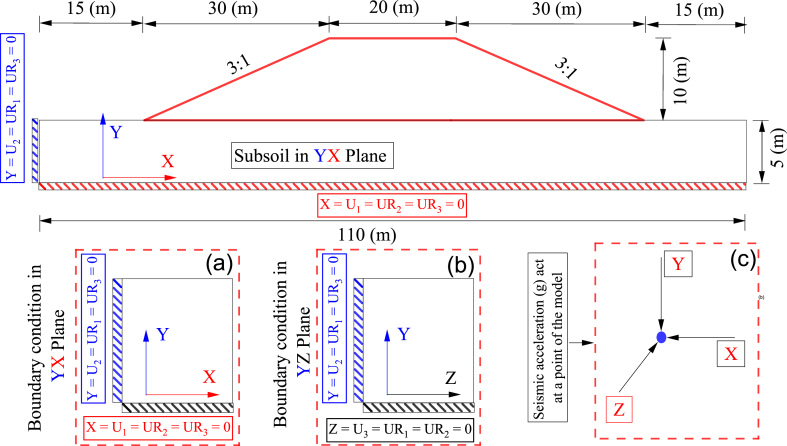
The impermeable embankment, subsoil, and boundary conditions, a) simulation of the boundary condition for the model in the YX plane, b) simulation of the boundary condition for the model in the YZ plane, c) applying seismic acceleration to each point of the model.

**Fig. 3:**
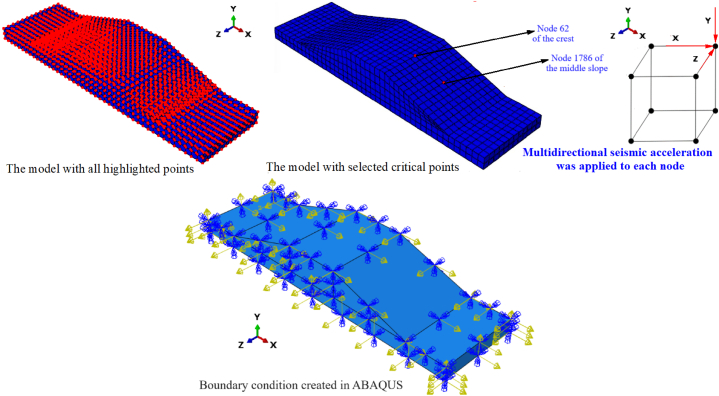
Mesh used in the numerical simulation, selected critical points of the model for analysis and prediction, and boundary condition created in ABAQUS.

Embankment earthquake response is related to geometry, mechanical properties, boundary conditions, and the nature of seismic acceleration [[4], [5], [6],^11^,^12^,^17^]. Fig. 1 shows the entire methodology used to estimate the displacement at the selected points of the embankment that was simulated from the polyurethane foam-sand mixture. NEFM was utilized to estimate displacement in the Y direction of the selected point in the model. The catastrophe theory was employed to predict the mechanism of differential displacement in multiple directions. The B-spline method was employed to simulate a nonlinear displacement curve. ANNs were used to validate results and predict displacement in the Y direction of the chosen points. Fig. 2 shows the embankment geometry and boundary condition. Seismic acceleration was applied to the model according to the boundary conditions. Fig. 3 shows the geometry of the embankment and subsoil with highlighted selected points for analysis as well as all of the points that are available in the model. Fig. 3 demonstrates the mechanism of applying multidirectional seismic acceleration to the crest and middle slope in numerical simulation.

The boundary condition of the model is explained in Fig. 2. According to the boundary condition, the seismic acceleration has been applied in three directions simultaneously at each point of the model. In addition, considering the displacement and the rotation of the model, the boundary condition in the YX plane and YZ plane has been changed. The tie size of 30 (m) was designed for the model. According to the geomorphology of a territory, the thickness of the embankment will be changed. Based on this concept, the constant thickness of the embankment was selected when the mechanical properties of the model were changed for each model.

The seismic acceleration history (g) in the 0°, 90°, and 360° were recorded in the earthquake station and were applied to the model. Based on the model's seismic acceleration history (g) and geometry, the boundary condition was simulated. The boundary condition created in ABAQUS has been depicted in Fig. 3. The seismic acceleration has been applied to the model on the five sides. In the Y direction, the seismic acceleration was applied in the base of the model, while in the X and Z directions, the seismic acceleration was applied on two opposite sides of the X and Z directions of the model. The same concepts were used for displacement and the rotation simulation of the model.

Longitudinal cracks occur in its crest due to earthquakes [25,26], and these longitudinal cracks mainly appear due to differential displacement on the crest and middle slope of the embankment model. Based on this concept, the main point of the investigated crest was node 62, and the main point of analysis in the middle slope was node 1786.

### 1.2 Catastrophe theory

Catastrophe theory is a set of mathematical methods for classifying a phenomenon that undergoes sudden, large changes. Table 1 illustrates Duffing's equation in catastrophe theory modeling [36]. In this study, the catastrophe progression value was compared with changing displacement in a point, considering strain, stress, and acceleration responses. Using catastrophe theory, the differential displacement of the selected point was predicted.

**Table 1:** The duffing's equation in catastrophe theory modeling [36].

Catastrophe	Control dimensions	Behavior dimensions	Function V	Equation number
Fold	1	1	$\frac{1}{3}\;x^{3}-a_{1}x$	(1)
Cusp	2	1	$\frac{1}{4}\;x^{4}-a_{1}x-\;\frac{1}{2}\;{a_{2}x}^{2}$	(2)
Swallowtail	3	1	$\frac{1}{5}\;x^{5}-a_{1}x-\;\frac{1}{2}\;{a_{2}x}^{2}-\;\frac{1}{3}\;{a_{3}x}^{3}$	(3)
Butterfly	4	1	$\frac{1}{6}\;x^{6}-a_{1}x-\;\frac{1}{2}\;{a_{2}x}^{2}-\;\frac{1}{3}\;{a_{3}x}^{3}-\;\frac{1}{4}\;{a_{4}x}^{4}$	(4)
Hyperbolic	3	2	$x^{3}+y^{3}+a_{1}x+a_{2}y+a_{3}xy$	(5)
Elliptic	3	2	$x^{3}-{xy}^{2}+a_{1}x+a_{2}y+a_{3}x^{2}+a_{3}y^{2}$	(6)
Parabolic	4	2	$x^{2}y+y^{4}+a_{1}x+a_{2}y+a_{3}x^{2}+a_{4}y^{2}$	(7)

For normalizing variables in simulating the catastrophe model Equations (8), (9)) were used. Equation (8) is appropriate for larger index values, and Equation (9) is appropriate for smaller index values [37].

(8) $$C=\frac{A}{A_{\max}}$$

(9) $$C=\frac{A_{\min}}{A}$$

Equations (10), (11)) were used to calculate the catastrophe progression value [38].

(10) x1=c112,x2=c113,x3=c314,x4=c415,….xn=cn1n+1

(11) $$X_{n}=\frac{x_{1\;}+x_{2\;}+x_{3\;}+\ldots +x_{n}}{n}$$

### 1.3 B-spline method

Schoenberg proposed the B-spline. The B-spline is a nonparametric density estimator for smoothing data sets and graphical presentation of a phenomenon [39]. In order to define a non-linear approximation operator for the B-spline method, equation (12) was suggested [40].

(12) $$Q_{P}^{H}\left(f\right)\left(x\right)=\sum_{n\in Z}L_{p}^{H}\left(f_{n},3\right)B_{p}\left(\frac{x}{h}-n\right)\;\text{With}\;p=2,3\text{.}$$

Where;

$Q_{P}^{H}\left(f\right)$ Non-linear operator

x Sign function

f Piecewise function

n∈Z Operators Lp

$L_{p}^{H}$ Coefficient for an estimate

$B_{p}\left(\frac{x}{h}-n\right)$ Partition of unity

p Piecewise polynomial of degree

h Interval jump value

Equations (13), (14)) were proposed for the modified B-spline method [40,41].

(13) $$H_{r}^{+}\left(x,y\right)=\frac{x+y}{2}\left(1-\;{\left|\frac{x-y}{x+y}\right|}^{r}\right)$$

(14) $$H_{r}^{+}\left(x,y\right)=\frac{2xy}{x+y}$$

The appropriate theoretical proofs were presented for proving the quality of accuracy in performing smooth zones during the B-spline method, and removal of Gibbs-like fluctuations adjacent discontinuities [40]. In this study, to predict the differential displacement mechanism at a selected point of the model, the B-spline method was applied to simulate catastrophe progression index value fluctuation.

## 2 Materials

**Fig. 4:**
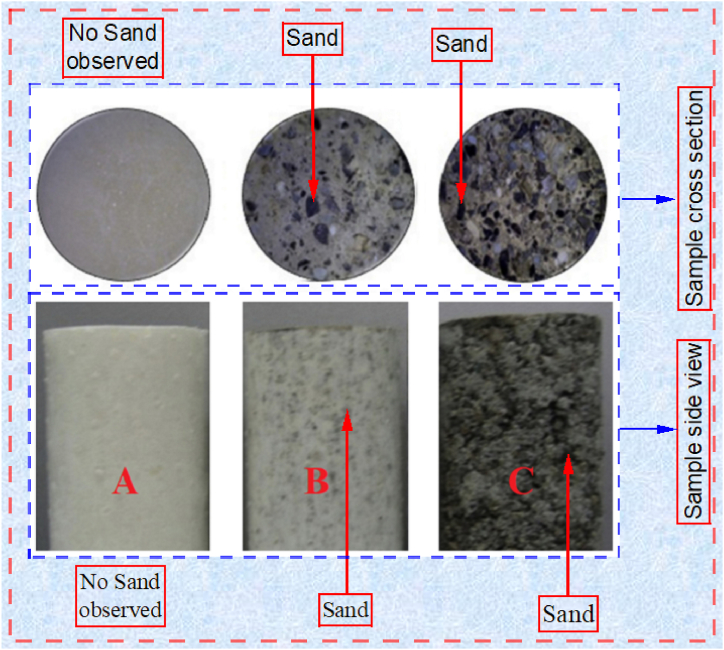
(A) Pure polyurethane foam, (B) 5 % polyurethane foam-sand, (C) 15 % polyurethane foam-sand [1].

The polyurethane foam and sand mixture was used in the embankment and subsoil simulation. In Table 2 [2], two types of composite materials are produced using 5 % and 15 % polyurethane foam mixed with sand. These two samples have different mechanical properties. Models 1 and 2 were simulated using polyurethane foam-sand 5 % and polyurethane foam-sand 15 %, respectively. Fig. 4 shows pure polyurethane foam, 5 % polyurethane foam-sand, and 15 % polyurethane foam-sand. The polyurethane foam-sand mixture was used in embankment and subsoil modeling. No sand was observed in sample (A). In samples (B) and (C), the black stains are from the sand concentration in the sample. The sand has not been distributed uniformly in the polyurethane foam-sand mixture. The influence of the permeability is not taken into account for the sake of simplicity, although it is well-known that the permeability of the foundation may affect the results.

**Table 2:** Mechanical properties of polyurethane foam-sand mixture [2].

Material	Modulus elasticity, E (MPa)	Unit weight, γ (kN/m^3^)	Poisson's ratio, *ν*
5 % Polyurethane foam - sand	150.8	1640	0.23
15 % Polyurethane foam - sand	10.1	461	0.04

### 2.1 Seismic data

**Fig. 5:**
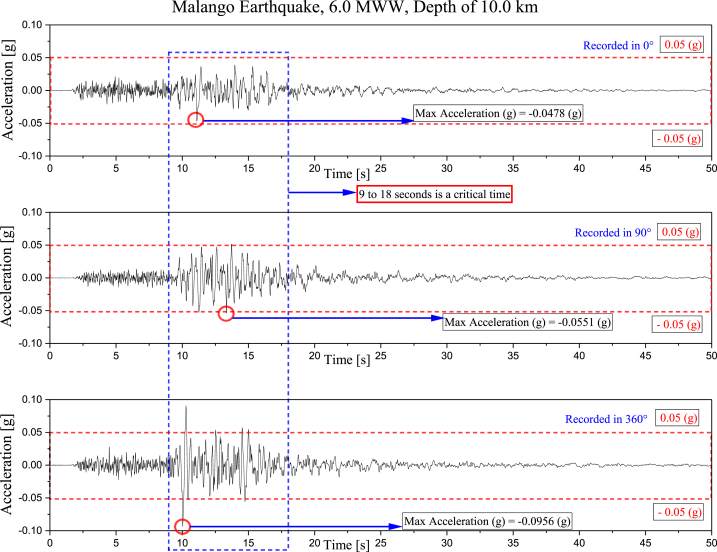
The acceleration history (g) in the 0°, 90°, and 360° [42].

**Fig. 6:**
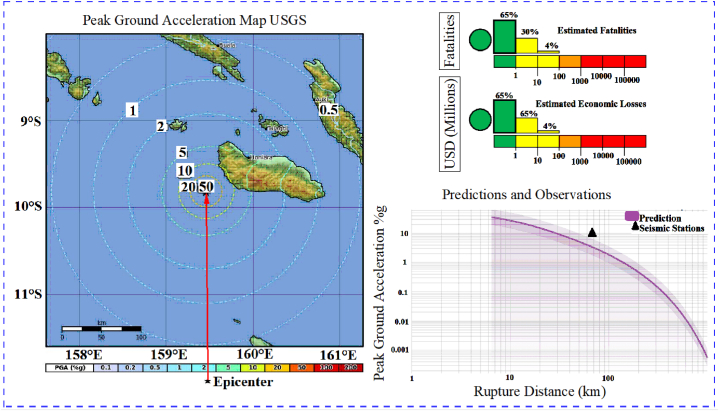
The earthquake characteristics [42].

Regarding the Malango earthquake, seismic data has been presented in Fig. 5, Fig. 6. This information was gathered by the Honiara Solomon Islands station, located 71.5 km away from the earthquake's epicenter. Fig. 5 highlights the acceleration characteristics in three directions: 0°, 90°, and 360°. It is essential to note that the acceleration directions between 9 and 18 s are particularly critical. Moreover, the acceleration range that prevails throughout the event is −0.05 (g) to 0.05 (g). Fig. 6 displays the Malango earthquake, which occurred at a depth of 10.0 km and had a magnitude of 6.0 MWW. It also displays the area affected by the earthquake. In addition, the maximum peak ground acceleration recorded at the earthquake station and predictions are also shown in Fig. 6 [42]. The USGS reported economic losses and fatalities due to this earthquake.

Appropriate simulated seismic loading on the model results in improved seismic design accuracy. The numerical simulation must apply a combination of multidirectional seismic loadings to the model. The domains of the combination of three acceleration directions control the stress, strain, and displacement developed in the model [12]. In recent years, the limited impact of multidirectional seismic loading on the model's seismic response has been investigated [[4], [5], [6],^11^,^12^,^43^]. In this work, a combination of seismic loading was applied to the model according to the boundary conditions of the model introduced in Fig. 2.

### 2.2 FEM

Abaqus software is used in industry and academia to solve problems that require finite elements. Abaqus relies heavily on the geometry of the model, the properties of the material, the boundary conditions, and the nature of the load. Whenever embankment and subsoil models are subjected to seismic loading, FEM can predict their behavior at any point along the embankment and subsoil. A finite element method was used to predict displacement, deformation, stress, and strain in the proposed model.

Using the ABAQUS, the 2990 and 2184 nodes and elements are designed for all models, respectively. The single style of the linear hexahedral elements of type C3D8R is designed. To improve simulation quality, the linear mesh has been designed in all areas of the model. When constructing the mesh for the numerical simulation, the warning and error were not seen. The mesh quality impacts the results of the numerical simulation that was checked in the simulation steps.

ABAQUS provides multifaceted simulated concepts to solve and non-destructively predict geotechnical problems. In this study, the results of the numerical simulation achieved by ABAQUS were compared with the outcome of the theoretical concept.

**Fig. 7:**
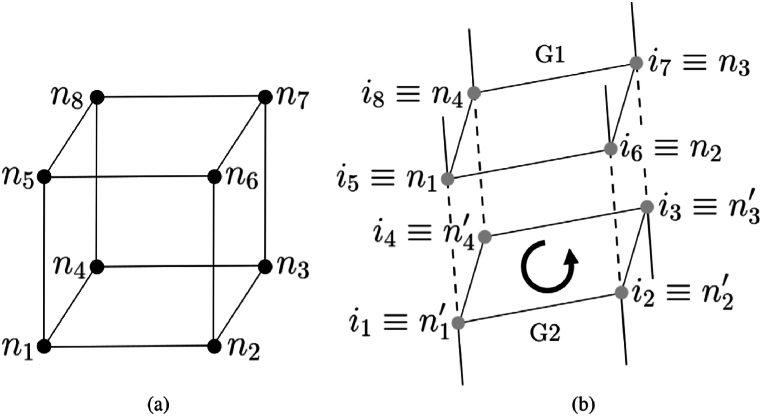
The number of nodes and interface elements [44].

Based on the model's shape, ABAQUS generates meshes that change in size. Fig. 7 [44] shows the number of nodes and interface elements. This study emphasizes the interaction between the embankment and the subsoil as the primary interface element.

By considering material characteristics, Biot theory [45,46] was used to develop the equation for fluid-saturated porous media. equations (15), (16), (17), (18)) express the model's nonlinear characteristics [47].

(15) $$L^{T}\sigma =\rho \ddot{u}+n\rho_{f}\left(\ddot{U}-\ddot{u}\right)$$

(16) $$\nabla P=nk_{f}^{-1}\left(\dot{U}-\dot{u}\right)+\rho_{f}\ddot{U}$$

(17) σ=Dε+αmP

(18) $$P=nQ\nabla^{T}\left(U-u\right)+\alpha Qm^{T}\varepsilon$$

Using the Galerkin method, equations (15), (16), (17), (18)) were applied to obtain equation (19), which is the u-U formed equation. This obtained the u-U formed equation used in finite elements [48]. For dynamic analysis of the 3D fluid-saturated element, ABAQUS was utilized based on the u-U formed equations [49].

(19) $$\left[\begin{array}{cc} \begin{array}{c} \overset{´}{m_{uu}}\\ 0 \end{array} & \begin{array}{c} 0\\ {\overset{´}{m}}_{UU} \end{array} \end{array}\right]\left[\begin{array}{c} \ddot{\bar{u}}\\ \ddot{\bar{U}} \end{array}\right]+\left[\begin{array}{cc} \begin{array}{c} \overset{´}{c_{uu}}\\ c_{uU}^{\prime T} \end{array} & \begin{array}{c} {\overset{´}{c}}_{uU}\\ {\overset{´}{c}}_{UU} \end{array} \end{array}\right]\left[\begin{array}{c} \dot{\bar{u}}\\ \dot{\bar{U}} \end{array}\right]+\left[\begin{array}{cc} \begin{array}{c} {\overset{´}{k}}_{uu}\\ k_{uU}^{\prime T} \end{array} & \begin{array}{c} {\overset{´}{K}}_{uU}\\ {\overset{´}{k}}_{UU} \end{array} \end{array}\right]\left[\begin{array}{c} \bar{u}\\ \bar{U} \end{array}\right]=\left[\begin{array}{c} {\bar{F}}_{u}\\ {\bar{F}}_{U} \end{array}\right]$$

Where;

$\overset{´}{m_{uu}}$ and ${\overset{´}{m}}_{UU}$ Mass submatrices

$\overset{´}{c_{uu}}$ , ${\overset{´}{c}}_{uU}$ , and, ${\overset{´}{c}}_{UU}$ Damping submatrices

${\overset{´}{k}}_{uu}$ , ${\overset{´}{K}}_{uU}$ and, ${\overset{´}{k}}_{UU}$ Stiffness submatrices

$\bar{u}$ Node displacement of soil

$\bar{U}$ Node displacement of fluid

${\bar{F}}_{u}$ Force of node related to displacement of the soil

${\bar{F}}_{U}$ Force of node related to the displacement of the fluid

An analysis of ground motions has been reported based on the occurrence of the 1957 San Francisco earthquake. It was adopted that the soil nonlinear behavior can be simulated through the equivalent linear method [50]. The equivalent linear was used with a complete understanding method to consider the soil nonlinearity behavior. The equivalent linear method is famous for solving engineering problems; it is adopted due to its distinguished characteristics of the well-defined concept, easy factors achievement, and considerable computational effectiveness [49]. The numerical simulation in the present work has been performed using the equivalent linear constitutive model, which is an effective approach.

### 2.3 ANNs

The study uses Levenberg-Marquardt back propagation training for Artificial Neural Networks (ANNs).

The training, validation, and testing process is essential to ANNs. Training sets are needed to calculate gradients and update weights and biases. Validation data is needed for cross-validation. Test set data is needed to determine the accuracy of ANNs. Equations (20), (21)) have been used [51] for ANNs.

(20) $$Q_{s}=B_{1}^{s}+\sum_{k=1}^{r}\left(w_{k,l}^{ho}\;\frac{2}{1+e^{{-2H}_{k}}}-1\right)$$

(21) $$H_{k}=B_{2}^{k}+\sum_{j=1}^{q}\left(w_{j,k}^{ih}\;.{\;I}_{j}\right)$$

Where;

$Q_{s}$ Normalized output

q Number of input.

$H_{k}$ Hidden neuron

r Number of hidden neurons

s Number of output.

$B_{1}^{s}$ Biases of the output neuron

$B_{2}^{k}$ Biases of hidden neuron

$w_{k,l}^{oh}$ Weights of connection between $O_{s}$ and $H_{k}$.

$w_{j,k}^{ih}$ Weights of connection between $I_{j}$ and $H_{k}$.

In assessing ANNs, mean squared error (MSE) and regression value R are determined using equations (22), (23)) [52]. In ANNs, MSE and R control the quality of prediction by considering obtained data by the FEM ($Y_{mea}$), mean obtained data by the FEM ($Y_{mea}$), and predicted data ($Y_{pre}$).Where;

$Y_{mea}$ Obtained data

$Y_{mean}$ Mean obtained data

$Y_{pre}$ Predicted data

(22) $$MSE=\frac{\sum_{i=1\;}^{n}{\left(y_{mea}\;-\;y_{pre}\right)}^{2}}{n}$$

(23) $$R^{2}=1-\frac{\sum_{i=1}^{n}{\left(Y_{mea}\;-\;Y_{pre}\right)}^{2}}{\sum_{i=1}^{n}{\left(Y_{mea}\;-\;Y_{mean}\right)}^{2}}$$

**Fig. 8:**
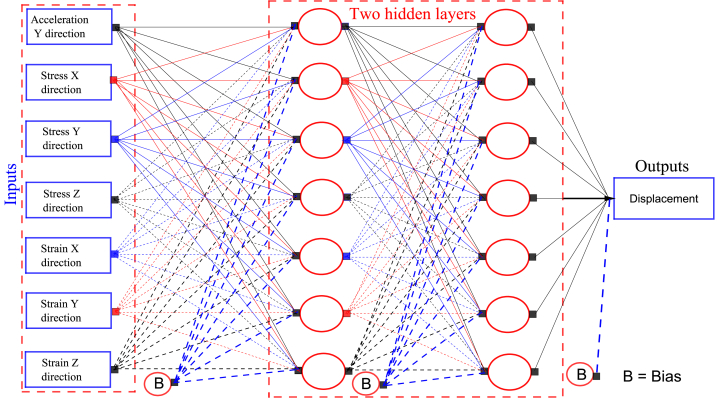
Simplified ANNs method with inputs, hidden layers and outputs.

Fig. 8 shows a simplified ANNs method with inputs, hidden layers, bias, and outputs. By considering a selected point for displacement prediction. At the selected point, the seismic acceleration response in the Y direction, stresses in X, Y, and Z directions, and strains in X, Y, and Z directions are input into ANNs. Two hidden layers have been used in the ANNs, and the output is displacement predictions. We used training, cross-validation, and testing with percentages of 70, 15, and 15 %, respectively.

## 3 Results and discussion

### 3.1 Displacement

**Fig. 9:**
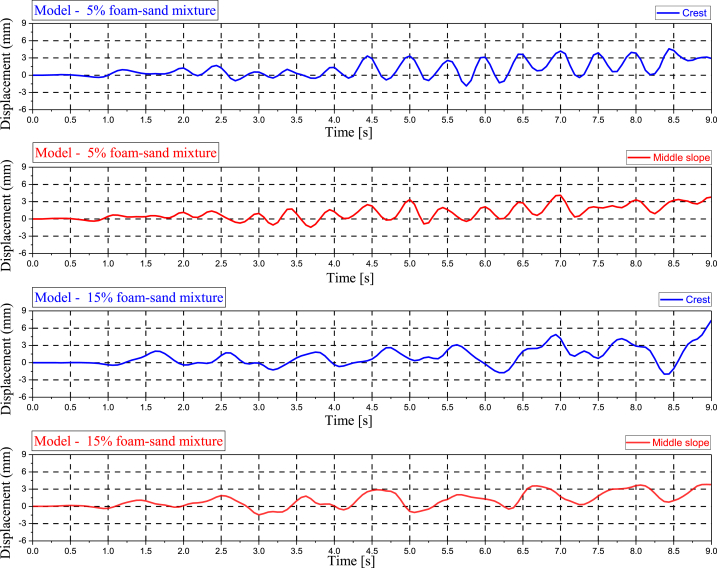
The displacement (mm) in the Y direction obtained NFEM.

Fig. 9 shows the displacement (mm) at the crest and middle slope of embankment models simulated from the 5 % polyurethane foam-sand mixture and the 15 % polyurethane foam-sand mixture. The displacement is in the Y direction. The displacement mechanism is shown as the percentage of the foam impact in nonlinear displacement at any point with the application of a seismic load. The highest displacement is observed at the crest when the embankment is modeled from a 15 % foam-sand mixture.

Polyurethane foam is a suitable material for producing polyurethane foam-sand mixtures as composite materials. The percentage of polyurethane foam and sand in the mixture process governs the mechanical properties of the polyurethane foam-sand mixture [1,2]. Other composite materials showed similar behavior. As a result of mixing tire chips with sand, a new composite material is created, which impacts the displacement and seismic load response structures. In addition, the polyurethane foam-sand mixture has an acceptable uniaxial compressive strength when subjected to static loads [1]. At any point in the model, as the polyurethane foam in the polyurethane foam-sand mixture increases, the displacement increases.

The flexibility of the polyurethane foam-sand mixture was associated with the material composite design. Increasing the amount of polyurethane in the polyurethane foam-sand mixing design increased the level of flexibility and deformation of the samples [1]. The polyurethane foam-sand mixing method has the potential to control the nonlinear displacements and vibrations of embankments and subsoils subject to seismic loads.

### 3.2 Acceleration and displacement

**Fig. 10:**
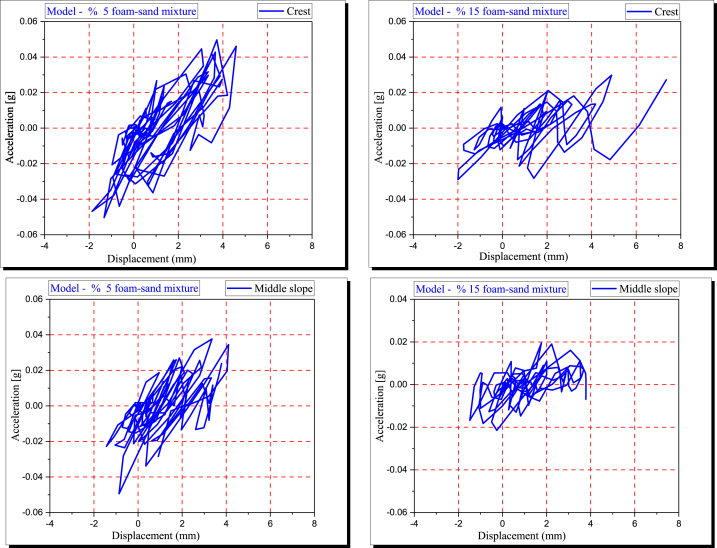
The acceleration (g) – displacement (mm) in the Y direction of the crest and middle slope.

Fig. 10 shows the acceleration (g)-displacement (mm) relationship in the Y direction of the crest and middle slope for two embankment models constructed from a 5 % polyurethane foam-sand mixture and a 15 % polyurethane foam-sand mixture. In both the crest and middle slope, the seismic acceleration of the model decreased as the percentage of polyurethane foam increased. Polyurethane foam considerably minimizes the acceleration transfer of the model. While displacement from point-to-point shows a different response. When the amount of polyurethane foam increased, displacement increased at some points, but the foam did not cause displacement to increase at other points. The geometry of the model, along with the mechanical properties of the model, control the nonlinear displacement mechanism of the model. A change in the differential displacement mechanism is associated with the percentage of polyurethane foam.

The displacement of the embankment at each point shows a different mechanism when the model is subjected to seismic acceleration. This is even when the applied seismic acceleration for each mode is the same [4]. In addition, the seismic response of the embankment is associated with the mechanical properties of the materials used to simulate the embankment and subsoil [53]. Higher acceleration fluctuations are observed with 5 % polyurethane foam in the polyurethane foam-sand mixture design. With increasing polyurethane foam percentage, the seismic response changed. The magnitudes of acceleration (g) and displacement (mm) at the crest and middle slope of any model vary. Different damping occurs for different models due to the fluctuation in acceleration (g) and displacement (mm).

The damping ratio increases with increasing foam content in the polyurethane foam-sand mixture [2], and the results of the present numerical simulation are in good agreement with the experimental results.

### 3.3 Stress and strain

**Fig. 11:**
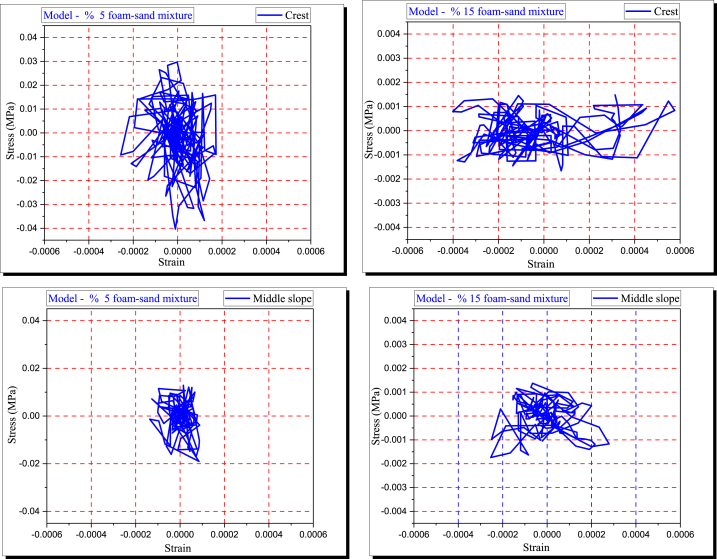
The stress (MPa) - strain for crest and middle slope considering foam-sand mixture percentage.

Fig. 11 shows how the stress-strain relationship in the embankment and subsoil models changes with respect to the polyurethane foam-sand mixture design. The model's seismic response is associated with the material's mechanical properties.

When optimization polymerization is used to make polyurethane, the modulus of elasticity, mode of failure, stiffness, and compressive and shear strengths of poorly graded sand are all changed [1]. A polyurethane foam-sand mixture's shear modulus decreases as foam content increases [2]. In this study, the polyurethane foam-sand mixture design controls the dynamic modulus of elasticity of the model.

### 3.4 Deformation and failure mode

**Fig. 12:**
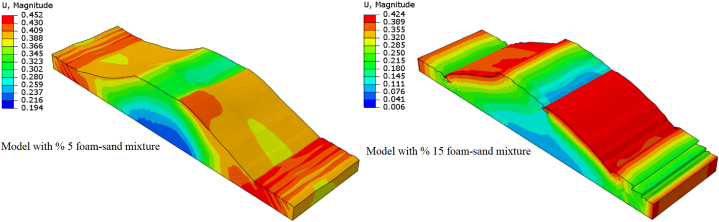
The 3D deformation of the model.

Fig. 12 shows the deformation at 0.5 s of applied seismic acceleration to the model. The model deformation changes with a polyurethane foam-sand mixture design.

The 5 % polyurethane foam-sand mixture exhibits higher shear resistance, while using 10 % or higher percentages of polyurethane leads to high-level polymerization, and this phenomenon is a reason for altering the mechanical properties of the polyurethane foam-sand mixture [1]. Because of the changing mechanical properties of the material, the model deformation was changed. Fig. 12 shows the polyurethane foam-sand mixture design governing the deformation pattern of the embankment and subsoil model.

There is a direct relationship between deformation and consolidation settlement in the embankment. In addition, on an embankment, settlement of the crest occurs due to embankment deformation [19]. At the crest of the embankment, the model with a 15 % polyurethane foam-sand mixture exhibits higher displacement and deformation compared to the model with a 5 % polyurethane foam-sand mixture.

In a saturated embankment zone, shear deformation occurs [19]. Using a polyurethane foam-sand mixture reduces the saturation of the embankment, and minimizes the shear deformation of the embankment.

The experiment showed large cracks on the saturated zone of the embankment. This phenomenon takes place due to horizontal tensile deformation in an embankment subjected to liquefaction [19]. The crack in the clayey crest of the embankment leads to deformation. The model deformation is reduced with an increasing clayey cohesive layer over the embankment crest [4]. While using a polyurethane foam-sand mixture, it minimizes crack occurrences due to the reduction of deformation on the embankment. Moreover, embankments constructed from polyurethane foam and sand will not liquefy.

### 3.5 Application of the catastrophe theory in differential displacement prediction

Based on the FEM, Table 3, Table 5, Table 7, Table 9 illustrate the seismic response of simulated embankments at the crest and middle slope by using 5 % polyurethane foam-sand mixture and 15 % polyurethane foam-sand mixture as simulation materials for embankments and subsoils. Stress and strain peaks in the X, Y, and Z directions, as well as acceleration in the Y direction, were used in the simulation of differential displacement using the catastrophe theory. Table 4, Table 6, Table 8, Table 10 illustrate the utility function values, according to catastrophe theory, of the simulated embankment at the crest and middle slope with a 5 % polyurethane foam-sand mixture and a 15 % polyurethane foam-sand mixture.

**Table 3:** Obtained data for predicting the seismic response of simulated embankment, the crest, in the model with 5 % polyurethane foam-sand mixture.

No	Peak stress (MPa) X direction, C_1_	Peak stress (MPa) Y direction, C_2_	Peak stress (MPa) Z direction, C_3_	Peak strain X direction, C_4_	Strain Y direction, C_5_	Strain Z direction, C_6_	Peaks Acceleration Direction Y, C_7_
1	0.02651	0.00117	−0.00274	1.69186E-4	−3.72739E-5	2.85231E-5	0.03005
2	−0.01817	−0.01471	1.3884E-4	1.21123E-4	−9.03888E-5	2.85231E-5	−0.0317
3	0.01757	−0.01134	−4.95216E-4	1.48049E-4	2.73759E-5	−3.23209E-5	0.02743
4	−0.02252	0.00902	0.0096	−1.47156E-4	1.09998E-4	−3.23209E-5	−0.02974
5	−0.02303	0.00604	0.00119	−8.01262E-7	−1.00844E-4	2.82143E-5	0.04393
6	0.0232	0.00155	0.00854	−1.33561E-4	−1.03263E-4	2.82143E-5	−0.03043
7	0.00126	−0.00658	−0.00384	1.44144E-4	−6.34119E-5	−2.41746E-5	0.04681
8	−0.00917	−0.0125	−0.00893	−1.75379E-4	8.63904E-5	−2.41746E-5	−0.0446
9	−0.02849	0.00147	−0.00763	1.7282E-4	1.22917E-4	−3.43897E-5	0.05036
10	0.02972	−0.00588	−0.00779	−2.35457E-5	1.362E-4	−3.43897E-5	−0.04275
11	−0.00541	0.01183	−0.00779	−2.56459E-4	1.15515E-4	−2.98203E-5	−0.04966
12	−0.04033	0.00235	−0.00199	−5.97565E-5	−3.04405E-5	−2.98203E-5	0.03133
13	−0.00901	0.00556	0.00749	1.15053E-4	4.77264E-6	−3.63867E-5	−0.04203
14	0.01713	−0.00805	−8.54077E-4	1.20175E-4	−1.02088E-4	−3.63867E-5	0.03219
15	−0.00604	0.01528	−0.00163	−1.45737E-4	9.54378E-5	−2.84548E-5	0.03635
16	−0.0367	0.00286	−0.00903	−2.25819E-4	9.92762E-5	2.13847E-5	−0.04605

**Table 4:** Utility function values according to catastrophe theory, the crest, in model with 5 % polyurethane foam-sand mixture.

No	C_1_	C_2_	C_3_	C_4_	C_5_	C_6_	C_7_	Displacement Y direction (mm)	Catastrophe progression value
1	0.65733	0.07657	−0.28542	0.00474	−0.12804	0.74973	0.91281	−0.97229	0.70964
2	0.45053	−0.9627	0.01446	0.00662	−0.0528	0.74973	−0.8653	−0.48704	0.70375
3	0.43566	−0.74215	−0.05159	0.00541	0.17434	−0.66164	1	−0.51873	0.72632
4	0.55839	0.59031	1	−0.00544	0.04339	−0.66164	−0.92233	−0.5131	0.78057
5	0.57104	0.39529	0.12396	−1	−0.04733	0.75794	0.6244	3.34136	0.79834
6	0.57525	0.10144	0.88958	−0.006	−0.04622	0.75794	−0.90141	−0.82005	0.72897
7	0.03124	−0.43063	−0.4	0.00556	−0.07526	−0.88459	0.58599	3.3462	0.66414
8	0.22737	−0.81806	−0.93021	−0.00457	0.05525	−0.88459	−0.61502	−0.93302	0.75363
9	0.70642	0.0962	−0.79479	0.00464	0.03883	−0.62183	0.54468	−1.86766	0.71821
10	0.73692	−0.38482	−0.81146	−0.03403	0.03504	−0.62183	−0.64164	3.12313	0.78514
11	0.13414	0.77421	−0.81146	−0.00312	0.04132	−0.71712	−0.55236	−1.33009	0.71701
12	1	0.1538	−0.20729	−0.01341	−0.15679	−0.71712	0.87552	3.66195	0.75774
13	0.22341	0.36387	0.78021	0.00696	1	−0.58771	−0.65263	4.20535	0.76738
14	0.42475	−0.52683	−0.08897	0.00667	−0.04675	−0.58771	0.85213	3.87453	0.69713
15	0.14976	1	−0.16979	−0.0055	0.05001	−0.75153	0.75461	3.97397	0.70208
16	0.90999	0.18717	−0.94063	−0.00355	0.04807	1	−0.59566	4.59714	0.76781

To calculate the catastrophe progression value absolute value was used in C_1_, C_2_, C_3_, C_4_, C_5_, C_6_, and C_7_ columns.

**Table 5:** Obtained data for predicting the seismic response of simulated embankment, middle slope, in the model with 5 % polyurethane foam-sand mixture.

No	Peak stress (MPa) X direction, C_1_	Peak stress (MPa) Y direction, C_2_	Peak stress (MPa) Z direction, C_3_	Peak strain X direction, C_4_	Strain Y direction, C_5_	Strain Z direction, C_6_	Peaks Acceleration Direction Y, C_7_
1	0.00791	−0.01062	−0.00645	6.16055E-5	−8.33337E-5	3.02059E-5	0.02357
2	0.00957	7.99378E-4	0.00584	7.5762E-5	−1.60386E-6	3.02059E-5	−0.02591
3	−3.47974E-4	−0.01357	0.00588	7.23498E-5	−1.00077E-4	−3.16075E-5	0.02276
4	−0.01608	0.00855	−0.00184	−6.15184E-5	−4.64899E-5	−3.16075E-5	−0.02579
5	0.0115	−0.01241	0.00506	−9.73596E-5	−4.49499E-5	2.80412E-5	−0.02015
6	−0.00256	−0.00527	9.34434E-5	8.69444E-5	6.74689E-5	−3.22033E-5	0.02157
7	−0.01903	0.00687	0.00665	7.67056E-6	−6.2108E-5	−3.22033E-5	−0.03765
8	−0.01903	−0.01128	−0.00665	−4.28334E-5	−3.93093E-5	2.9671E-5	0.04943
9	−0.00133	0.00265	−0.00665	−4.28334E-5	7.16945E-5	2.9671E-5	−0.02205
10	−0.00225	−0.00402	−0.00313	−3.1809E-5	7.14556E-5	2.55059E-5	−0.02701
11	−0.00225	0.01327	−0.00653	7.61481E-5	6.44189E-5	2.55059E-5	0.0199
12	−0.01417	0.00245	−0.00604	7.61481E-5	−9.15438E-5	2.63779E-5	−0.02561
13	0.00863	−0.00516	0.0046	−1.00663E-4	−9.15438E-5	2.63779E-5	0.01963
14	0.00464	−0.01	0.00425	6.68708E-5	3.74946E-5	3.12591E-5	−0.03448
15	0.00464	0.00837	0.00709	3.56552E-5	6.54703E-5	3.12591E-5	0.03377
16	−0.01479	9.90868E-4	0.00408	−9.9127E-5	9.87796E-5	3.29328E-5	0.02847

**Table 6:** Utility function values according to catastrophe theory, middle slope, in the model with 5 % polyurethane foam-sand mixture.

No	C_1_	C_2_	C_3_	C_4_	C_5_	C_6_	C_7_	Displacement Y direction (mm)	Catastrophe progression value
1	0.41566	−0.78261	−0.90973	0.12451	0.01925	0.8444	0.83284	−0.38887	0.81048
2	0.50289	0.05891	0.8237	0.10125	1	0.8444	−0.75762	−0.71792	0.80364
3	−0.01829	−1	0.82934	0.10602	0.01603	−0.80696	0.86248	−1.04371	0.74022
4	−0.84498	0.63007	−0.25952	−0.12469	0.0345	−0.80696	−0.76115	−1.43586	0.80808
5	0.60431	−0.91452	0.71368	−0.07879	0.03568	0.90959	−0.97419	2.48145	0.83226
6	−0.13452	−0.38836	0.01318	0.08822	−0.02377	−0.79203	0.91006	−0.2025	0.6489
7	−1	0.50626	0.93794	1	0.02582	−0.79203	−0.52138	3.35565	0.88769
8	−1	−0.83125	−0.93794	−0.17908	0.0408	0.85962	0.39713	−0.86249	0.86995
9	−0.06989	0.19528	−0.93794	−0.17908	−0.02237	0.85962	−0.89025	−0.42244	0.71894
10	−0.11823	−0.29624	−0.44147	−0.24114	−0.02245	1	−0.72677	2.0983	0.72429
11	−0.11823	0.97789	−0.92102	0.10073	−0.0249	1	0.98643	2.872	0.7838
12	−0.74461	0.18055	−0.8519	0.10073	0.01752	0.96694	−0.7665	4.10286	0.78469
13	0.45349	−0.38025	0.6488	−0.0762	0.01752	0.96694	1	2.10104	0.77111
14	0.24383	−0.73692	0.59944	0.11471	−0.04278	0.81595	−0.56932	2.28774	0.77432
15	0.24383	0.6168	1	0.21513	−0.0245	0.81595	0.58129	3.32328	0.78931
16	−0.77719	0.07302	0.57546	−0.07738	−0.01624	0.77448	0.6895	3.38862	0.7417

To calculate the catastrophe progression value absolute value was used in C_1_, C_2_, C_3_, C_4_, C_5_, C_6_, and C_7_ columns.

**Table 7:** Obtained data for predicting the seismic response of simulated embankment, the crest, in model with 15 % polyurethane foam-sand mixture.

No	Peak stress (MPa) X direction, C_1_	Peak stress (MPa) Y direction, C_2_	Peak stress (MPa) Z direction, C_3_	Peak strain X direction, C_4_	Strain Y direction, C_5_	Strain Z direction, C_6_	Peaks Acceleration Direction Y, C_7_
1	0.00458	−0.00126	−0.00127	−2.0622E-4	8.32844E-5	−1.20582E-4	−0.01362
2	−0.0026	0.00124	−0.00128	4.51708E-4	−1.32067E-4	−1.20582E-4	−0.01581
3	−0.00215	4.32762E-4	−0.00128	−2.53706E-4	3.22011E-5	−1.28941E-4	0.01775
4	0.00442	−0.00166	0.0018	−2.09678E-4	−9.47789E-6	−1.28941E-4	−0.01548
5	0.00316	0.00123	0.0018	4.35396E-4	−8.0501E-5	−1.57823E-4	0.01449
6	0.00338	−0.00129	0.00103	3.35882E-4	6.50316E-6	−1.57823E-4	−0.01462
7	−0.00293	0.0015	0.0011	−2.85396E-4	1.42941E-4	−1.15596E-4	0.01353
8	−8.90553E-4	−0.00143	0.0012	−9.00075E-5	1.44871E-4	−1.15596E-4	−0.02977
9	−0.00111	0.00131	0.0012	−1.11541E-4	−1.3994E-4	−1.26044E-4	0.02822
10	−0.0016	−4.66063E-4	0.00171	−1.5821E-4	1.34068E-4	−1.26044E-4	−0.02044
11	−0.00408	0.00243	0.00171	−4.01306E-4	2.14022E-4	−1.20058E-4	0.02134
12	0.00585	0.00196	−7.99275E-4	5.76289E-4	2.00343E-4	−1.20058E-4	−0.01354
13	−8.59968E-4	0.0015	−0.00126	−9.50033E-5	1.58497E-4	−1.62799E-4	−0.02111
14	−0.00162	−0.00243	−0.00126	−1.57931E-4	−2.25576E-4	−1.62799E-4	0.02892
15	−0.00392	0.00221	0.00146	−3.81541E-4	2.02494E-4	−1.35357E-4	−0.01813
16	0.00336	4.51714E-4	0.00146	3.36657E-4	3.3887E-5	−1.35357E-4	0.01775

**Table 8:** Utility function values according to catastrophe theory, the crest, in model with 15 % polyurethane foam-sand mixture.

No	C_1_	C_2_	C_3_	C_4_	C_5_	C_6_	C_7_	Displacement Y direction (mm)	Catastrophe progression value
1	0.78291	−0.51852	−0.70556	−0.43646	0.07808	−0.95865	−0.99339	−0.01515	0.87126
2	−0.44444	0.51029	−0.71111	0.19926	−0.04924	−0.95865	−0.85579	−0.45183	0.81263
3	−0.36752	0.17809	−0.71111	−0.35477	0.20195	−0.8965	0.76225	1.99205	0.80244
4	0.75556	−0.68313	1	−0.42927	−0.68614	−0.8965	−0.87403	−0.40134	0.92876
5	0.54017	0.50617	1	0.20673	−0.08078	−0.73244	0.93375	−0.12773	0.83814
6	0.57778	−0.53086	0.57222	0.26797	1	−0.73244	−0.92544	−0.20335	0.87927
7	−0.50085	0.61728	0.61111	−0.31538	0.0455	−1	1	−1.26539	0.83353
8	−0.15223	−0.58848	0.66667	−1	0.04489	−1	−0.45448	1.83744	0.80486
9	−0.18974	0.53909	0.66667	−0.80695	−0.04647	−0.91711	0.47945	−0.66412	0.80151
10	−0.2735	−0.1918	0.95	−0.56891	0.04851	−0.91711	−0.66194	2.60808	0.7888
11	−0.69744	1	0.95	−0.22429	0.03039	−0.96283	0.63402	3.1115	0.86598
12	1	0.80658	−0.44404	0.15618	0.03246	−0.96283	−0.99926	−1.74588	0.85661
13	−0.147	0.61728	−0.7	−0.94741	0.04103	−0.71005	−0.64093	4.89458	0.80347
14	−0.27692	−1	−0.7	−0.56992	−0.02883	−0.71005	0.46784	2.01669	0.82143
15	−0.67009	0.90947	0.81111	−0.23591	0.03212	−0.85401	−0.74628	4.17887	0.85588
16	0.57436	0.18589	0.81111	0.26736	0.19191	−0.85401	0.76225	−1.99858	0.82136

To calculate the catastrophe progression value absolute value was used in C_1_, C_2_, C_3_, C_4_, C_5_, C_6_, and C_7_ columns.

**Table 9:** Obtained data for predicting the seismic response of simulated embankment, middle slope, in the model with 15 % polyurethane foam -sand mixture.

No	Peak stress (MPa) X direction, C_1_	Peak stress (MPa) Y direction, C_2_	Peak stress (MPa) Z direction, C_3_	Peak strain X direction, C_4_	Strain Y direction, C_5_	Strain Z direction, C_6_	Peaks Acceleration Direction Y, C_7_
1	−9.43321E-4	0.00116	−0.00121	−9.48783E-5	9.67157E-5	1.39872E-4	−0.00898
2	4.24338E-4	−0.0014	−0.00135	4.13159E-5	1.17397E-4	1.39872E-4	0.01669
3	4.96579E-4	9.9769E-4	−0.00135	1.25079E-4	−1.46189E-4	−1.71517E-4	0.01109
4	−0.00132	9.9769E-4	−0.00167	5.17123E-5	1.72573E-5	−1.71517E-4	−0.01949
5	−0.00135	−2.17938E-4	−0.00167	−1.33482E-4	1.72573E-5	1.2516E-4	0.01296
6	−2.05959E-4	9.82032E-4	0.00115	−1.34035E-4	−1.09314E-4	1.2516E-4	−0.01076
7	−2.05959E-4	−0.00163	0.00115	−2.33098E-5	1.06291E-4	1.35805E-4	0.01269
8	0.00214	−3.54916E-4	−0.00129	−2.33098E-5	−1.51522E-4	1.35805E-4	−0.01899
9	−0.00153	−0.00117	−0.00129	2.15977E-4	−1.83592E-4	1.52507E-4	0.01823
10	0.0014	0.00137	0.00138	−1.54025E-4	−1.21714E-4	1.52507E-4	0.00968
11	0.00198	0.00188	0.00138	1.40912E-4	1.46053E-4	1.52233E-4	−0.01095
12	−0.00247	0.00135	0.00176	1.99914E-4	1.98488E-4	1.52233E-4	0.02146
13	−0.00252	−8.75742E-4	0.00176	−2.50101E-4	1.44513E-4	−1.25568E-4	−0.01605
14	0.00276	−8.75742E-4	−0.00131	−2.53869E-4	−9.62395E-5	−1.25568E-4	0.01181
15	−7.31307E-4	−5.04239E-4	−0.00143	2.79176E-4	−9.62395E-5	1.189E-4	0.01473
16	0.00116	−0.00106	−0.00143	1.16217E-4	−5.99046E-5	1.189E-4	−0.0111

**Table 10:** Utility function values according to catastrophe theory, middle slope, in the model with 15 % polyurethane foam -sand mixture.

No	C_1_	C_2_	C_3_	C_4_	C_5_	C_6_	C_7_	Displacement Y direction (mm)	Catastrophe progression value
1	−0.34178	0.61702	−0.6875	−0.24568	0.17843	0.85006	−1	−0.38343	0.83273
2	0.15375	−0.74468	−0.76705	0.56418	0.147	0.85006	0.53805	−0.14434	0.82217
3	0.17992	0.53069	−0.76705	0.18636	−0.11805	−0.69323	0.80974	1.85444	0.7868
4	−0.47826	0.53069	−0.94886	0.45076	1	−0.69323	−0.46075	−1.48011	0.88536
5	−0.48913	−0.11592	−0.94886	−0.17463	1	0.94998	0.6929	−1.01527	0.83245
6	−0.07462	0.52236	0.65341	−0.17391	−0.15787	0.94998	−0.83457	1.76423	0.7697
7	−0.07462	−0.86702	0.65341	−1	0.16236	0.87552	0.70764	0.32237	0.82904
8	0.77536	−0.18879	−0.73295	−1	−0.11389	0.87552	−0.47288	−0.61421	0.8525
9	−0.55435	−0.62234	−0.73295	0.10793	−0.094	0.77964	0.49259	2.88114	0.81699
10	0.50725	0.72872	0.78409	−0.15134	−0.14179	0.77964	0.92769	−1.07812	0.8452
11	0.71739	1	0.78409	0.16542	0.11816	0.78104	−0.82009	2.0094	0.8753
12	−0.89493	0.71809	1	0.1166	0.08694	0.78104	0.41845	−0.4921	0.85998
13	−0.91304	−0.46582	1	−0.0932	0.11942	−0.9469	−0.5595	3.5471	0.85383
14	1	−0.46582	−0.74432	−0.09182	−0.17932	−0.9469	0.76037	0.28172	0.86197
15	−0.26497	−0.26821	−0.8125	0.08349	−0.17932	1	0.60964	3.6897	0.77266
16	0.42029	−0.56383	−0.8125	0.20057	−0.28808	1	−0.80901	0.72861	0.84794

To calculate the catastrophe progression value absolute value was used in C_1_, C_2_, C_3_, C_4_, C_5_, C_6_, and C_7_ columns.

**Fig. 13:**
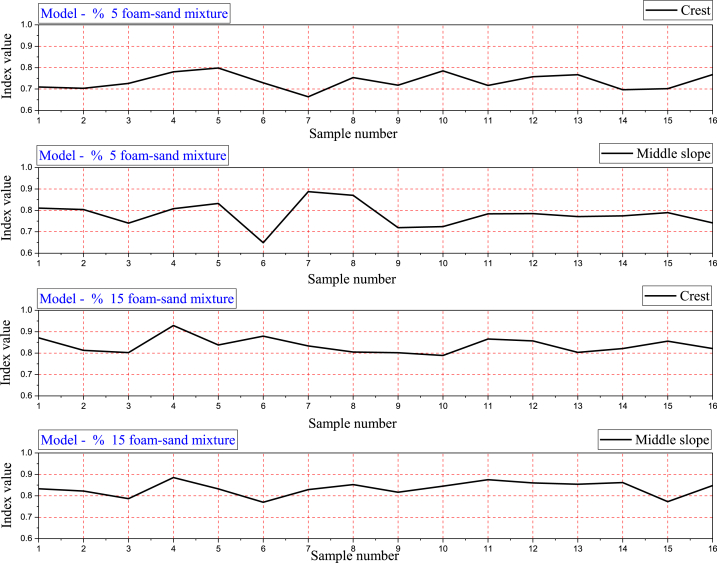
The catastrophe progression index value for predicting the differential displacement mechanism of the crest and middle slope critical nodes.

The mechanism of the differential displacement in the multidirectional at the crest and middle slope on the embankment model was predicted using catastrophic theory. Fig. 13 shows the catastrophe progression index value for the nonlinear displacement at the crest and middle slope for models 1 and 2. Nonlinear displacement fluctuation is reduced with an increasing percentage of polyurethane in the polyurethane foam-sand mixture. In addition, the catastrophic theory, as well as FEM results, confirm that with an increasing percentage of polyurethane in the polyurethane foam-sand mixture, the vibration mechanism is reduced.

Based on the catastrophe progression value and seismic factor safety of the embankment, it can be seen that the catastrophe theory can predict the earthquake response of the embankment.

It has been observed that the catastrophe progression and value of the safety factor follow similar fluctuation patterns [37]. Catastrophe theory is a qualitative method with quantitative outcomes. It can solve discontinuity problems [36]. The catastrophe theory confirms that models 1 and 2 differ in differential displacement at any point.

### 3.6 B-spline applied in catastrophe progression index value

**Fig. 14:**
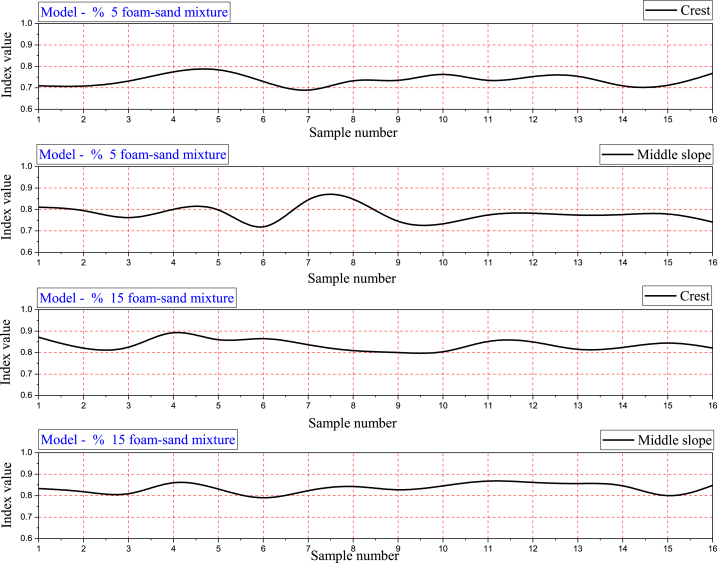
The catastrophe progression index value for predicting differential displacement mechanism by applying the SP-line method.

In applying the catastrophe theory, we have selected 16 peaks from around 300 data points produced by FEM for each stress, strain, and acceleration response. However, the catastrophe theory confirms the results obtained by FEM in explaining nonlinear displacement in the crest and middle slope. To integrate catastrophe theory results, B-spline needs to be applied to the catastrophe progression index value for clarification of the results. Fig. 13 shows the catastrophe progression index value for predicting the differential displacement mechanism at the crest and middle slope of the embankment. Fig. 14 shows the catastrophe progression index value for predicting the differential displacement mechanism by using the B-spline method. In terms of vibration mechanism, catastrophe theory and the B-spline result in a differential displacement mechanism that is similar to FEM-derived displacement in the Y direction.

As a result of using the classical quasi-interpolation operator, B-splines eliminate oscillations near discontinuity jumping. In order to confirm the theoretical results, numerical experiments were presented [40]. This work uses the B-spline method to minimize the number of calculations required to determine the differential displacement mechanism at the crest and middle slope in the embankment based on catastrophe theory. Therefore, the proposed method improves the engineering decision-making process for the seismic design of embankments built with polyurethane foam-sand mixtures.

### 3.7 Statistical analysis and prediction

**Fig. 15:**
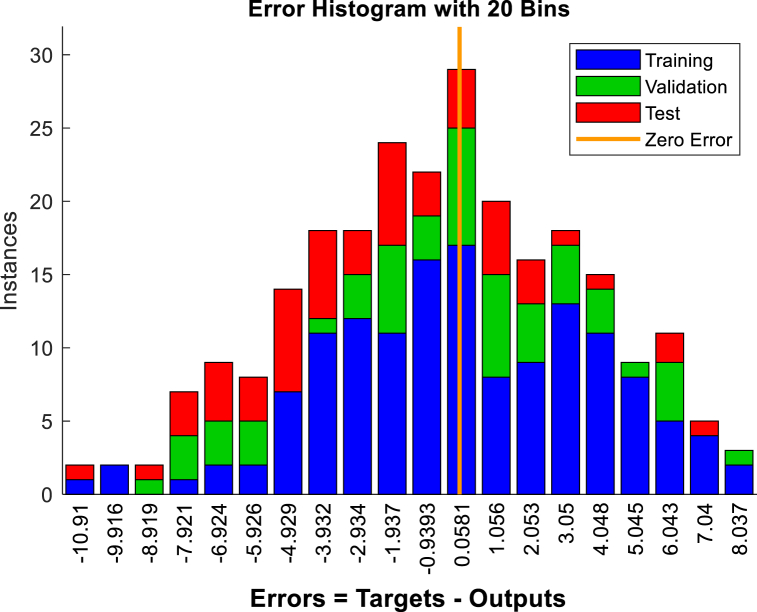
The displacement (mm) prediction of the crest at the model with the 5 % polyurethane foam-sand mixture.

To predict displacement in the crest and middle slope of the embankment ANNs were performed based on Levenberg-Marquardt algorithms. Two hidden layers were selected for the ANNs. The data presented in Table 3, Table 5, Table 7, Table 9, were used in performing the ANNs. As shown in Fig. 15, Fig. 17, Fig. 19, Fig. 21, histograms show the displacement range predicted by the model simulated from the 5 % polyurethane foam-sand mixture and the 15 % polyurethane foam-sand mixture at the crest, and the middle slope. The predicted displacement was in the direction of Y.

**Fig. 16:**
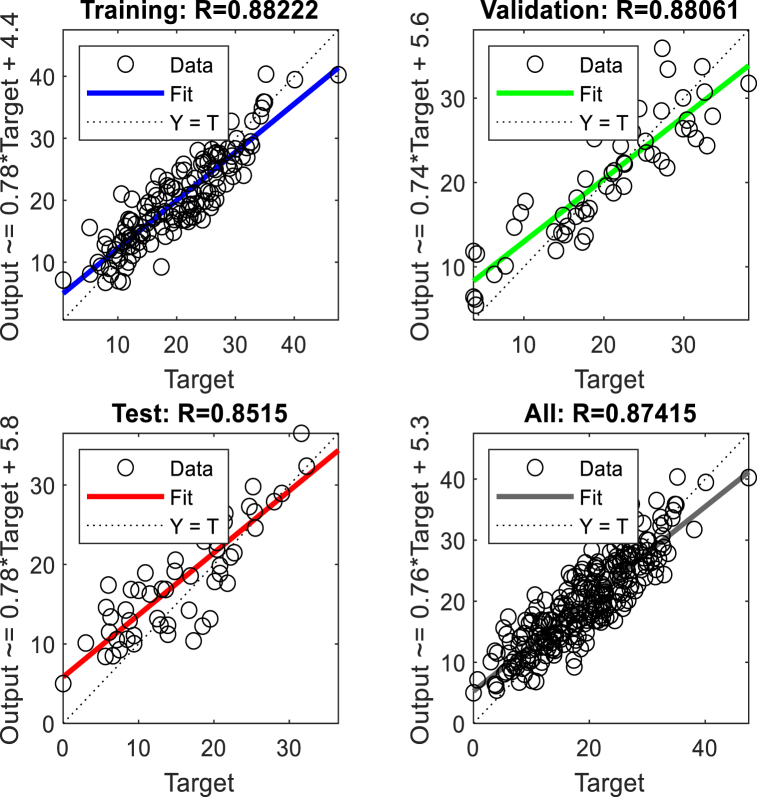
The regression analysis of the crest at model with the 5 % polyurethane foam-sand mixture.

Fig. 16, Fig. 18, Fig. 20, Fig. 22 show regression analysis, which indicates displacement prediction accuracy. Table 11, Table 12, Table 13, Table 14 present the mean squared error (MSE) and R2 results. Based on the regression analysis results, the ANNs made an acceptable prediction (see Fig. 23).

**Fig. 17:**
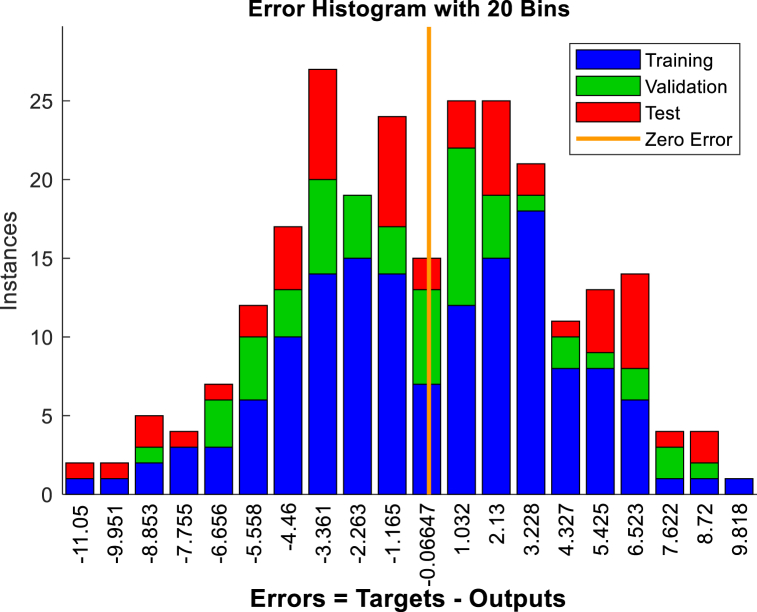
The displacement (mm) prediction of the middle slope at the model with the 5 % polyurethane foam-sand mixture.

**Fig. 18:**
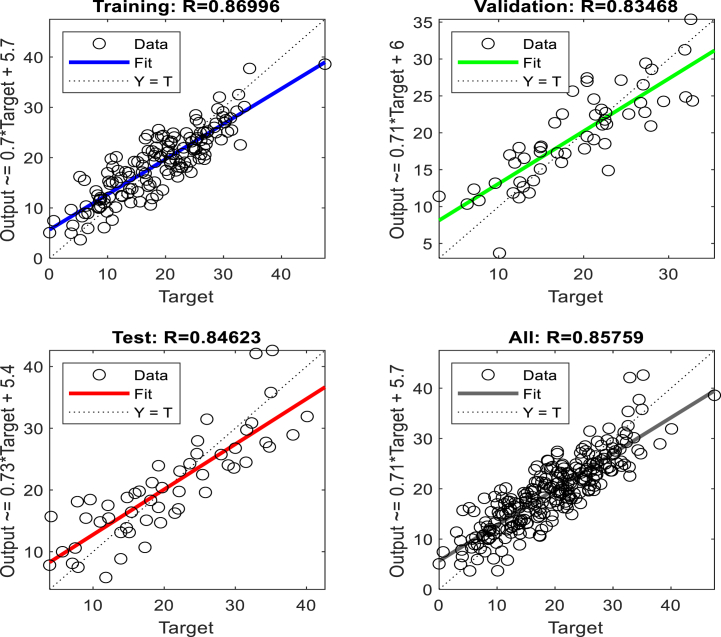
The regression analysis of the middle slope at the model with the 5 % polyurethane foam-sand mixture.

**Fig. 19:**
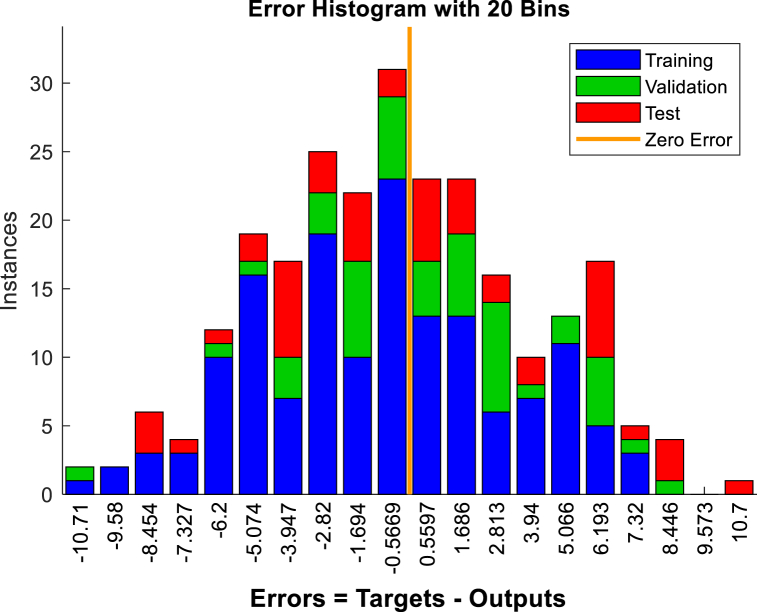
The displacement (mm) prediction of the crest at the model with the 15 % polyurethane foam-sand mixture.

**Fig. 20:**
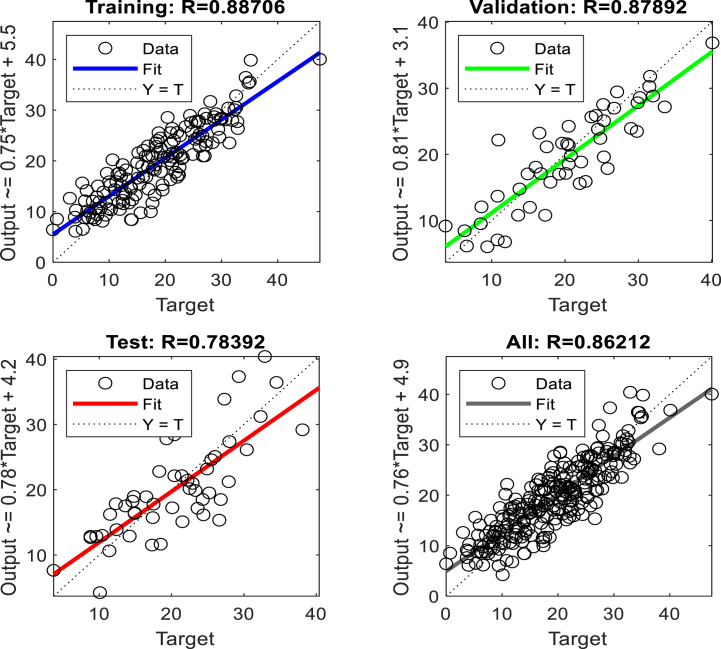
The regression analysis of the crest at model with the 15 % polyurethane foam-sand mixture.

**Fig. 21:**
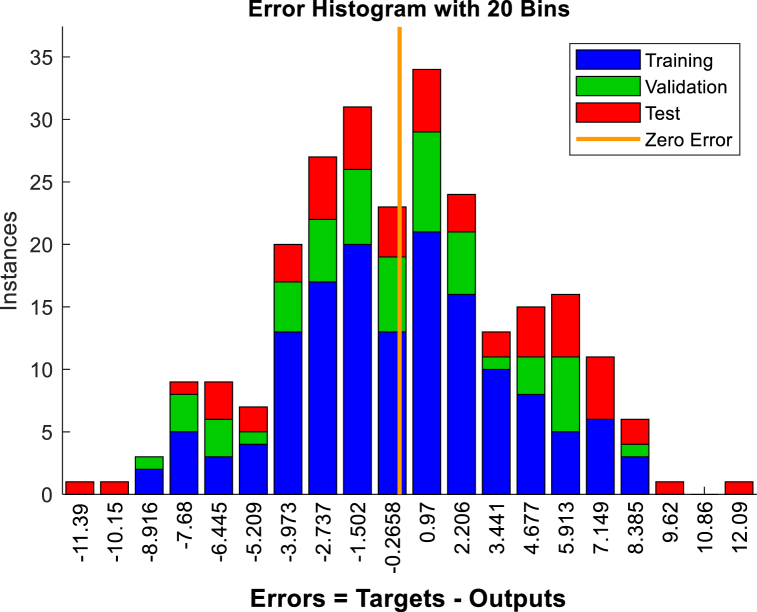
The displacement (mm) prediction of the middle slope at the model with the 15 % polyurethane foam-sand mixture.

**Fig. 22:**
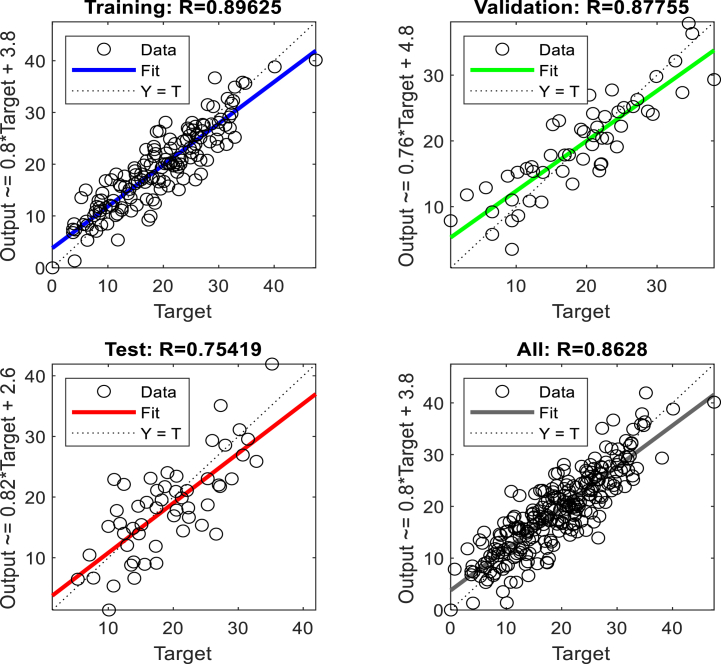
The regression analysis of the middle slope at the model with the 15 % polyurethane foam-sand mixture.

**Fig. 23:**
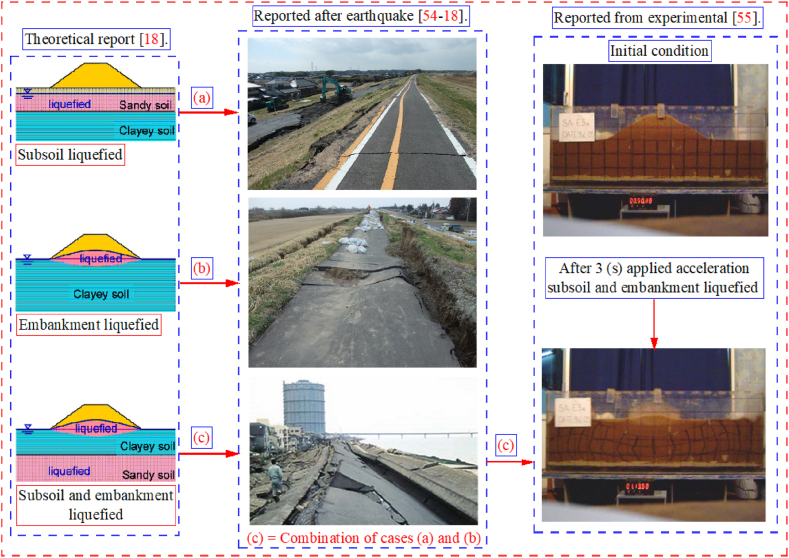
The different liquefaction cases [18,54,55].

**Table 11:** R^2^ and MSE results, the crest, at model with the 5 % polyurethane foam-sand mixture.

-	Training	Validation	Test	Number of layers in ANNs
R^2^	0.88222	0.88061	0.8515	2
MSE	14.79	17.31	21.57	2

**Table 12:** R^2^ and MSE results, the middle slope, at the model with the 5 % polyurethane foam -sand mixture.

-	Training	Validation	Test	Number of layers in ANNs
R^2^	0.86996	0.83468	0.84623	2
MSE	16.85	16.12	25.5	2

**Table 13:** R^2^ and MSE results, the crest, at the model with the 15 % polyurethane foam-sand mixture.

-	Training	Validation	Test	Number of layers in ANNs
R^2^	0.88706	0.87892	0.78392	2
MSE	16.97	15.24	24.28	2

**Table 14:** R^2^ and MSE results, the middle slope, at the model with the 15 % polyurethane foam-sand mixture.

-	Training	Validation	Test	Number of layers in ANNs
R^2^	0.89625	0.87755	0.75419	2
MSE	14.84	16.81	28.33	2

### 3.8 A rationale for using polyurethane foam sand

The Yodo River embankment collapsed during the 1995 Kobe earthquake due to liquefaction [54]. Throughout the world, sandy embankments and subsoil liquefy. Liquefaction may begin in the embankment, subsoil, or both simultaneously as shown in Fig. 23, [54-18]. It has been found that 70 % of liquefaction occurs in the subsoil, 10 % in the embankment, and 20 % in both at the same time [18]. Experimentally, liquefaction occurs simultaneously in loose saturated sandy embankments and subsoils [55]. The liquefaction of road embankments and other infrastructure such as telecommunication systems was investigated to understand failure mechanisms caused by the liquefaction phenomenon [[56], [57], [58]]. Because of this engineering problem, the polyurethane foam-sand mixture has been used in this study to simulate embankments and subsoils.

The main important part of the embankment collapse appeared due to a lack of sufficient tensile strength. To integrate the prediction and classification of the seismic response of the embankment the Multivariate Adaptive Regression Splines (MARS) models, support vectors machines (SVM), Gaussian's process regressions (GPR), and other mathematical modeling could be used to improve the research outcome [[59], [60], [61]]. As available in the literature, Soft mathematical methods with multilayer frameworks have been applied to solve several problems. For future investigation, these soft mathematical methods are applicable in solving new scientific problems [[62], [63], [64], [65], [66], [67], [68]]. These soft mathematical methods with complex networks are applicable in vast branches of science.

## 4 Conclusions

A polyurethane foam-sand mixture was used to simulate the embankment. The seismic response of embankment and subsoil was studied. The nonlinear displacement at selected points of the embankment was evaluated using Artificial Neural Networks (ANNs), catastrophe theory, and the B-spline method. Also, the deformation of the embankment and subsoil was assessed using different percentages of polyurethane foam-sand mixture. The following points were observed after applying different theoretical concepts to the results of the nonlinear numerical simulation.

• The model construct of a 15 % polyurethane foam-sand mixture has higher flexibility than a 5 % polyurethane foam-sand mixture.
• The displacement, strain, and deformation of the embankment-subsoil model increased when more polyurethane foam was used in a polyurethane foam-sand mixture design.
• In the polyurethane foam-sand mixture design, the stress/strain response of the model was affected by the percentage of polyurethane foam. The stress is affected by the level of the polyurethane foam.
• The seismic loading response changes with increasing polyurethane foam percentage.
• The displacement at each point in the model follows a different mechanism. The outcome of catastrophic theory is the same as the results of FEM. This confirms that with an increasing percentage of polyurethane in the polyurethane foam-sand mixture, the vibration mechanism is reduced. By applying a B-spline to the simulation of differential displacement according to catastrophe theory, we were able to achieve the best quality of the simulation.
• A suitable polyurethane foam-sand mixture design needs to be designed based on seismic stability analysis, the geometry of the embankment, and external loads applied to the embankment.
• It was found that most stabilized sandy subsoil collapsed due to liquefaction. By using the presented method in constructing the embankment, the chance for liquefaction has been eliminated.
• Polyurethane foam-sand mixing can be used to control the nonlinear displacement and vibration of embankments and subsoils subjected to seismic loads.
• From an economic point of view, in a territory to solve the problem of suitable construction materials availability in constructing impermeable embankment, using a polyurethane foam-sand mixture is the cost-effective and appropriate method of construction.
• The limitation of the work is related to the experimental simulation in the large-scale model. Based on cost-effective numerical simulation and the impossibility of large seismic simulation, the proposed method in the present study provided the best solution for realizing seismic resistance prediction of the embankment made of the polyurethane foam-sand mixture.

## Ethics approval

We have read and understood the journal's policies, and we believe that neither the manuscript nor the study violates any of these.

## Conflict of interest

The authors declare that they have no conflicts of interest.

## Data availability

Data are contained within this manuscript.

## Funding

Abu Dhabi University, Abu Dhabi, 10.13039/100016565UAE, funded this study as part of an internal project.

## Consent for publication

All the authors have approved the manuscript for submission and publication.

**Table:** Nomenclatural

$Q_{P}^{H}\left(f\right)$	Non-linear operator
*x*	Sign function
*f*	Piecewise function
n ∈ Z	Operators Lp
$L_{p}^{H}$	Coefficient for an estimate
$B_{p}\left(\frac{x}{h}-n\right)$	Partition of unity
p	Piecewise polynomial of degree
*h*	Interval jump value
$Y_{mea}$	Obtained data
$Y_{mean}$	Mean obtained data
$Y_{pre}$	Predicted data
$Q_{s}$	Normalized output
q	Number of input
$H_{k}$	Hidden neuron
r	Number of hidden neurons
s	Number of output
$B_{1}^{s}$	Biases of output neuron
$B_{2}^{k}$	Biases of hidden neuron
$w_{k,l}^{oh}$	Weights of connection between $O_{s}$ and $H_{k}$
$w_{j,k}^{ih}$	Weights of connection between $I_{j}$ and $H_{k}$
$\overset{´}{m_{uu}}$ and ${\overset{´}{m}}_{UU}$	Mass submatrices
$\overset{´}{c_{uu}}$ , ${\overset{´}{c}}_{uU}$ , and, ${\overset{´}{c}}_{UU}$	Damping submatrices
${\overset{´}{k}}_{uu}$ , ${\overset{´}{K}}_{uU}$ and, ${\overset{´}{k}}_{UU}$	Stiffness submatrices
$\bar{u}$	Node displacement of soil
$\bar{U}$	Node displacement of fluid
${\bar{F}}_{u}$	Force of node related to displacement of the soil
${\bar{F}}_{U}$	Force of node related to displacement of the fluid

## CRediT authorship contribution statement

**Omer Mughieda:** Writing – review & editing, Writing – original draft, Supervision, Methodology, Investigation, Conceptualization. **Abdoullah Namdar:** Software. **Wen Nie:** Methodology, Formal analysis, Data curation.

## Declaration of competing interest

The authors declare that they have no known competing financial interests or personal relationships that could have appeared to influence the work reported in this paper.
